# Influenza A(H3N2) Virus in Swine at Agricultural Fairs and Transmission to Humans, Michigan and Ohio, USA, 2016 

**DOI:** 10.3201/eid2309.170847

**Published:** 2017-09

**Authors:** Andrew S. Bowman, Rasna R. Walia, Jacqueline M. Nolting, Amy L. Vincent, Mary Lea Killian, Michele M. Zentkovich, Joshua N. Lorbach, Sarah E. Lauterbach, Tavis K. Anderson, C. Todd Davis, Natosha Zanders, Joyce Jones, Yunho Jang, Brian Lynch, Marisela R. Rodriguez, Lenee Blanton, Stephen E. Lindstrom, David E. Wentworth, John Schiltz, James J. Averill, Tony Forshey

**Affiliations:** The Ohio State University, Columbus, Ohio, USA (A.S. Bowman, J.M. Nolting, M.M. Zentkovich, J.N. Lorbach, S.E. Lauterbach);; US Department of Agriculture, Ames, Iowa, USA (R.R. Walia, A.L. Vincent, M.L. Killian, T.K. Anderson, J. Schiltz);; Centers for Disease Control and Prevention, Atlanta, Georgia, USA (C.T. Davis, N. Zanders, J. Jones, Y. Jang, B. Lynch, M.R. Rodriguez, L. Blanton, S.E. Lindstrom, D.E. Wentworth);; Michigan Department of Agriculture and Rural Development, Lansing, Michigan, USA (J.J. Averill);; Ohio Department of Agriculture, Reynoldsburg, Ohio (T. Forshey)

**Keywords:** animals, disease outbreaks, influenza A virus, zoonoses, livestock, public health, swine, RNA viruses, animal population groups, agricultural fair, Ohio, Michigan, United States, respiratory infections

## Abstract

In 2016, a total of 18 human infections with influenza A(H3N2) virus occurred after exposure to influenza-infected swine at 7 agricultural fairs. Sixteen of these cases were the result of infection by a reassorted virus with increasing prevalence among US swine containing a hemagglutinin gene from 2010–11 human seasonal H3N2 strains.

Influenza A virus infects many animal species. Zoonotic transmission allows for the introduction of novel influenza A virus strains to the human population, which has the potential to cause the next influenza pandemic. Swine exhibitions at agricultural fairs have emerged as a source for amplification of swine-lineage influenza A virus; these unique swine–human interfaces have generated most human infections with variant influenza A virus in the United States (*1*). 

During July–August 2016, outbreaks of variant H3N2 virus (H3N2v) were reported in Ohio and Michigan, and 18 zoonotic influenza A virus infections were detected (*2*). All persons identified with H3N2v infections during these outbreaks reported swine exposure while attending >1 of 7 fairs in Ohio or Michigan. We examined the role of exhibition swine in the transmission of this reassortant influenza A virus, which contained a hemagglutinin gene from 2010–11 human seasonal H3N2 strains.

## The Study

Active influenza A virus surveillance among exhibition swine occurred during summer 2016 at 101 agricultural fairs across the midwestern United States; pigs were sampled at the end of exhibition irrespective of clinical signs of respiratory disease (*3*). Samples obtained using nasal swabs or nasal wipes were stored in viral transport medium at −80°C (*4*,*5*). Upon notification from the state animal health official, samples collected from pigs at fairs associated with H3N2v cases were screened for influenza A virus with real-time reverse transcription PCR, and positive samples were inoculated for virus isolation as previously described (*6*). The genomes of 1 or 2 isolates per fair were sequenced, and the nucleotide sequences were deposited into GenBank (*7*). Nucleotide sequences of the H3N2v viruses detected in humans were deposited in the GISAID database (Technical Appendix). 

We used MAFFT version 7.222 (*8*) to align sequences and manually corrected them in MEGA7 (*9*). We inferred maximum-likelihood trees by using IQ-TREE version 1.4.3 under a general time reversible plus gamma distribution plus invariant sites evolutionary model (*10*), and assessed branch support using an ultrafast bootstrap approximation with 1,000 replicates (*11*). We visualized and annotated trees using MEGA7.

We found that 7 fairs in Ohio (n = 4) and Michigan (n = 3) were associated with human H3N2v cases during July–August 2016. Of those, 6 (fairs A–F) were participating in the active influenza A virus swine surveillance program. We also included a diagnostic lab submission for a pig with respiratory disease at the seventh fair (fair G) in this study.

We sampled 161 pigs across the 7 fairs, and isolated H3N2 virus from >1 pig at each fair. Based on virus isolation data from Fairs A–F (Table 1), the average prevalence of influenza A–infected swine in these fairs was 77.5% (individual fair range: 60%–90%), indicating extensive influenza A virus amplification within the swine at each of these fairs. However, widespread influenza-like illness among the swine was reported at only 2 of the fairs (Fairs A and E), suggesting that subclinical influenza A infections in pigs remain a threat to public health (3).

**Table 1:** Influenza A virus rRT-PCR and virus isolation test results of samples from active surveillance among swine at agricultural fairs, Michigan and Ohio, USA, 2016*

Fair	ILI among swine reported	No. swine sampled	No. (%) positive	
			rRT-PCR	Isolation
A	Yes	20	20 (100)	18 (90)
B	No	20	17 (85)	17 (85)
C	No	20	20 (100)	18 (90)
D	No	20	18 (90)	14 (70)
E	Yes	20	20 (100)	18 (90)
F	No	20	15 (75)	12 (60)

*Nasal swab or nasal wipe samples were collected from swine at the end of the fair. ILI, influenza-like illness; rRT-PCR, real-time reverse transcription PCR.

We sampled 161 pigs across the 7 fairs, and isolated H3N2 virus from >1 pig at each fair. Virus isolation data from fairs A–F (Table 1) indicated that the average prevalence of influenza A–infected swine in these fairs was 77.5% (individual fair range 60%–90%), indicating extensive influenza A virus amplification within the swine at each of these fairs. However, widespread influenza-like illness among swine was reported at only 2 of the fairs (fairs A and E), suggesting that subclinical influenza A infections in pigs remain a threat to public health (*3*).

**Figure 1:**
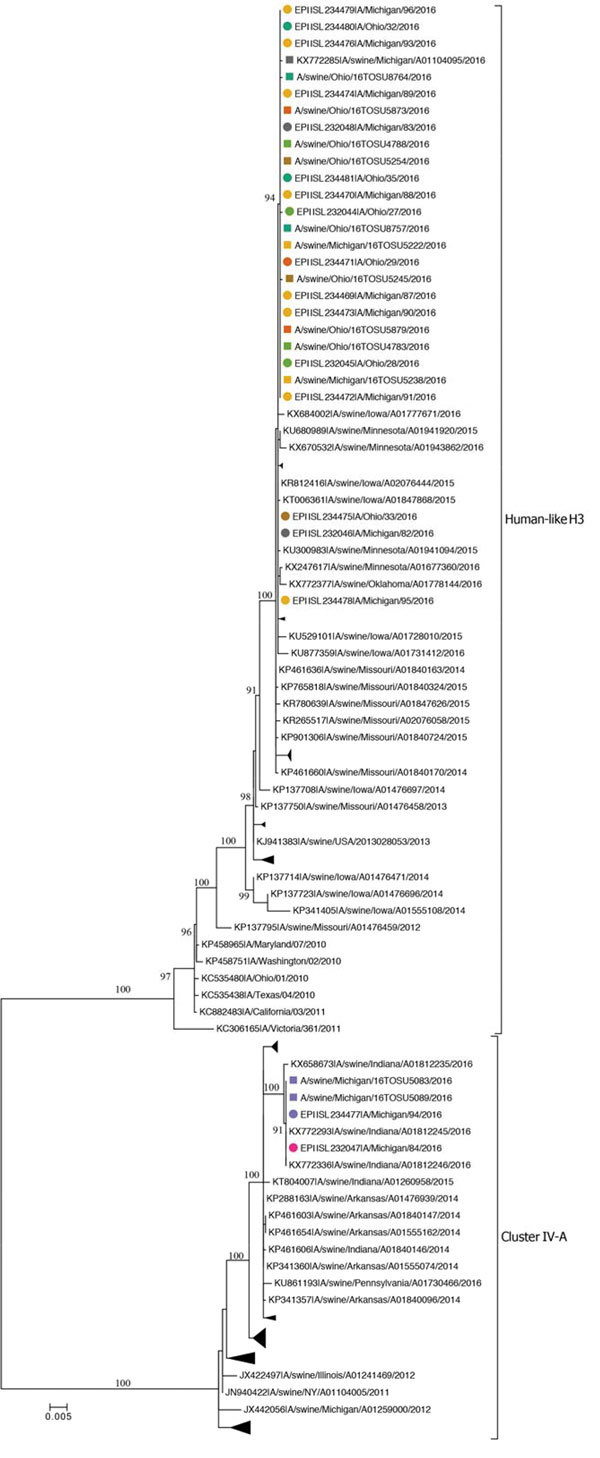
Phylogenetic relationships inferred for subtype H3 hemagglutinin genes of 2 distinct lineages (indicated on the right of the tree) from influenza A viruses isolated from swine and humans at agricultural fairs in Ohio and Michigan, USA, 2016. Isolates recovered are shown as squares for swine and circles for humans; colors indicate the fair attended. Scale bar indicates nucleotide substitutions per site; collapsed clades within each lineage are monophyletic clades of swine H3 HA genes.

A fair-by-fair comparison of the influenza A virus genomes sequenced from human H3N2v cases and isolates from swine provided strong molecular evidence of zoonotic influenza A virus transmission. The viruses recovered from swine were nearly identical to viruses identified in humans, and human virus gene segment sequences were nested within monophyletic swine virus clades. We identified 2 distinct H3 lineages in the pigs and humans across the implicated fairs (Figure 1). An influenza A virus from the well-established H3 cluster IV-A., found in the pigs at fair C, was responsible for 2 (11.1%) human cases. This cluster IV-A H3N2 genome belonged to the previously described H3 genotype 1 (Table 2) and was similar to the viruses responsible for the H3N2v infections detected in 2011–2013 (*12*). The influenza A virus detected in swine at the 6 fairs associated with the remaining 16 (88.9%) human H3N2v cases was a relatively new H3 lineage in swine. The HA gene of this virus descended from the human seasonal H3N2 virus circulating in 2010–11, which has since reassorted with enzootic swine influenza A viruses to produce novel viruses in the US swine herd (*13*). The other 7 gene segments in this human-like H3 reassortant virus were of the same lineages as those segments found in the cluster IV-A virus (Table 2).

**Table 2:** H3N2 genotypes identified in influenza A viruses detected in exhibition swine at agricultural fairs associated with 16 human variant H3N2 cases, Michigan and Ohio, USA, 2016*

Genotype	PB2	PB1	PA	HA	NP	NA	M	NS
H3 genotype 1, n = 2	trig	trig	trig	Swine cluster IV-A	trig	2002	pdm	trig
Human-like H3, n = 11	trig	trig	trig	Human-like H3	trig	2002	pdm	Trig

*Genomic constellations of 13 influenza A virus isolates from swine are organized by the 8 gene segments of the influenza A virus genome with HA categorized as derived from the 2010–11 human seasonal H3N2 virus (human-like H3) or the established swine lineage H3N2 (cluster IV-A). The 6 internal gene segments (PB2, PB1, PA, NP, M, NS) are classified as originating from either the 1998 triple-reassortant internal gene (trig) or influenza A(H1N1)pdm09 (pdm). All NA genes were descendants of the 2002 N2 lineage common among North American swine (*12*). HA, hemagglutinin; M, matrix; NA, neuraminidase; NP, nucleoprotein; NS, nonstructural protein; PA, polymerase acidic; PB, polymerase basic.

Irrespective of the fair of origin, the genomic sequences of all 11 human-like H3N2 virus isolates from swine were >99.89% identical to each other, demonstrating clonal expansion of 1 virus across 2 states. This pattern of virus dissemination within the exhibition swine population was a hallmark of the 2012 fair season, when 306 H3N2v human cases were reported (*6*).

**Figure 2:**
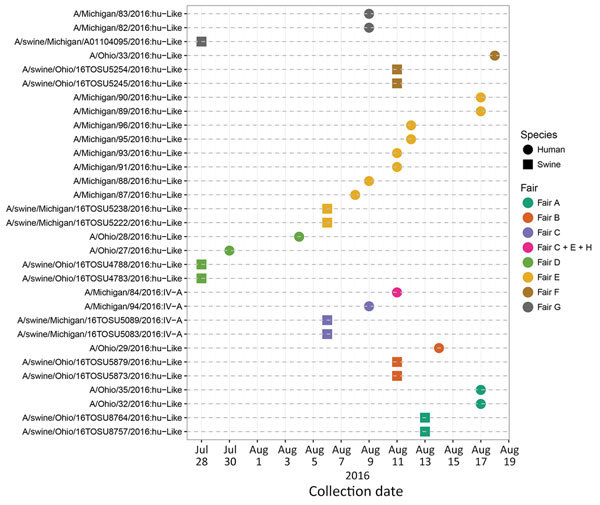
Timeline of detection of human and swine influenza A virus isolates at agricultural fairs in 2016. Isolates recovered are shown as squares for swine and circles for humans; colors indicate the fair attended. One person was exposed to pigs at 3 fairs (C, E, and H). Fair H is an eighth location not described in this study.

Influenza A virus was detected in pigs at each fair at least 1 day before each H3N2v virus infection was detected in humans (Figure 2). The observed lag time between the collection of human and swine samples is probably a function of the timing for active surveillance in swine (i.e., swine are sampled at the end of the fair), whereas specimens were collected from humans when they showed symptoms of influenza-like illness (Figure 2). Retrospective investigations of infections in the swine from these fairs would not have been possible if the pig sampling relied on protocols triggered by the detection of H3N2v virus cases in humans because fairs typically run for 1 week and infected swine would have been dispersed before sampling could have occurred.

## Conclusions

Variant influenza infections in humans continue to occur through contact with exhibition swine; often, the cases are in swine exhibitors with close and prolonged swine exposure. The concurrent detection of genetically identical influenza A viruses from exhibition swine across 2 states illustrates the rapidity with which this virus, and potentially other pathogens, can move within the highly mobile exhibition swine population. In addition to the zoonotic risks of influenza A virus, this pattern serves as a warning of possible dissemination of other emerging or high-consequence diseases in swine. Management practices common in the exhibition swine industry (i.e., frequent exhibition and relaxed biosecurity) facilitate the rapid dissemination of influenza virus across a large geographic landscape (*14*). Collaboration between animal and public health officials facilitated this investigation. Methods to control intraspecies and interspecies influenza virus transmission during swine shows have been outlined by the National Association of State Public Health Veterinarians (http://nasphv.org/Documents/Influenza_Transmission_at_Swine_Exhibitions_2016.pdf).

The recovery of human-like H3 influenza A viruses from exhibition swine supports previous studies demonstrating that the US commercial swine herd can serve as an influenza A reservoir for the much smaller exhibition swine population, which is more accessible to humans. Within the US commercial herd, the proportion of H3 isolates containing human-like H3 nearly doubled to 46% in spring and summer 2016 (data not shown). Whereas human-like H3s have been circulating, reassorting, and becoming more prevalent in the commercial swine population since 2012, introduction and expansion of the human-like H3 reassortant influenza A viruses in exhibition swine facilitated documented zoonoses from this genotype. The path traversed by this human-like H3, from initial introduction from humans to swine until the zoonotic transmission events of 2016, demonstrates how novel viruses can be generated and maintained in animal populations and, subsequently, can infect humans through specific ecologic niches like swine exhibitions or live-animal markets (*15*). Therefore, continued surveillance in swine populations is imperative for detecting novel influenza A viruses that threaten swine and human health.

Technical Appendix Nucleotide sequences of influenza A H3N2v viruses detected in humans.

## References

[1] Jhung MA, Epperson S, Biggerstaff M, Allen D, Balish A, Barnes N, et al. Outbreak of variant influenza A(H3N2) virus in the United States. Clin Infect Dis. 2013;57:1703–12. 10.1093/cid/cit649 24065322 PMC5733625

[2] Schicker RS, Rossow J, Eckel S, Fisher N, Bidol S, Tatham L, et al. Outbreak of influenza A(H3N2) Variant virus infections among persons attending agricultural fairs housing infected swine—Michigan and Ohio, July–August 2016. MMWR Morb Mortal Wkly Rep. 2016;65:1157–60. 10.15585/mmwr.mm6542a1 27787493

[3] Bowman AS, Nolting JM, Nelson SW, Slemons RD. Subclinical influenza virus A infections in pigs exhibited at agricultural fairs, Ohio, USA, 2009-2011. Emerg Infect Dis. 2012;18:1945–50. 10.3201/eid1812.121116 23171654 PMC3557874

[4] Nolting JM, Szablewski CM, Edwards JL, Nelson SW, Bowman AS. Nasal wipes for influenza A virus detection and isolation from swine. J Vis Exp. 2015; (106):e53313. 26709840 10.3791/53313 PMC4692777

[5] Edwards JL, Nelson SW, Workman JD, Slemons RD, Szablewski CM, Nolting JM, et al. Utility of snout wipe samples for influenza A virus surveillance in exhibition swine populations. Influenza Other Respi Viruses. 2014;8:574–9. 10.1111/irv.12270 25043408 PMC4161620

[6] Bowman AS, Nelson SW, Page SL, Nolting JM, Killian ML, Sreevatsan S, et al. Swine-to-human transmission of influenza A(H3N2) virus at agricultural fairs, Ohio, USA, 2012. Emerg Infect Dis. 2014;20:1472–80. 10.3201/eid2009.131082 25148572 PMC4178388

[7] Bowman AS, Sreevatsan S, Killian ML, Page SL, Nelson SW, Nolting JM, et al. Molecular evidence for interspecies transmission of H3N2pM/H3N2v influenza A viruses at an Ohio agricultural fair, July 2012. Emerg Microbes Infect. 2012;1:e33. 10.1038/emi.2012.33 26038404 PMC3630945

[8] Katoh K, Standley DM. MAFFT multiple sequence alignment software version 7: improvements in performance and usability. Mol Biol Evol. 2013;30:772–80. 10.1093/molbev/mst010 23329690 PMC3603318

[9] Kumar S, Stecher G, Tamura K. MEGA7: Molecular Evolutionary Genetics Analysis version 7.0 for bigger datasets. Mol Biol Evol. 2016;33:1870–4. 10.1093/molbev/msw054 27004904 PMC8210823

[10] Nguyen LT, Schmidt HA, von Haeseler A, Minh BQ. IQ-TREE: a fast and effective stochastic algorithm for estimating maximum-likelihood phylogenies. Mol Biol Evol. 2015;32:268–74. 10.1093/molbev/msu300 25371430 PMC4271533

[11] Minh BQ, Nguyen MA, von Haeseler A. Ultrafast approximation for phylogenetic bootstrap. Mol Biol Evol. 2013;30:1188–95. 10.1093/molbev/mst024 23418397 PMC3670741

[12] Kitikoon P, Nelson MI, Killian ML, Anderson TK, Koster L, Culhane MR, et al. Genotype patterns of contemporary reassorted H3N2 virus in US swine. J Gen Virol. 2013;94:1236–41. 10.1099/vir.0.051839-0 23695819

[13] Rajão DS, Gauger PC, Anderson TK, Lewis NS, Abente EJ, Killian ML, et al. Novel reassortant human-like H3N2 and H3N1 influenza A viruses detected in pigs are virulent and antigenically distinct from swine viruses endemic to the United States. J Virol. 2015;89:11213–22. 10.1128/JVI.01675-15 26311895 PMC4645639

[14] Nelson MI, Stucker KM, Schobel SA, Trovão NS, Das SR, Dugan VG, et al. Introduction, evolution, and dissemination of influenza A viruses in exhibition swine in the United States during 2009 to 2013. J Virol. 2016;90:10963–71. 10.1128/JVI.01457-16 27681134 PMC5110178

[15] Choi MJ, Torremorell M, Bender JB, Smith K, Boxrud D, Ertl JR, et al. Live animal markets in Minnesota: a potential source for emergence of novel influenza A Viruses and interspecies transmission. Clin Infect Dis. 2015;61:1355–62. 10.1093/cid/civ618 26223994 PMC4599395

