# Supplementary material for: Influenza A(H3N2) Virus in Swine at Agricultural Fairs and Transmission to Humans, Michigan and Ohio, USA, 2016
Source: Emerg Infect Dis. 2017 Sep;23(9):1551–5. doi: 10.3201/eid2309.170847 (PMC5572863; doi:10.3201/eid2309.170847)
Supplement: Technical Appendix — Nucleotide sequences of influenza A H3N2v viruses detected in humans. [file 17-0847-Techapp-s1.pdf]

Article DOI: https://doi.org/10.3201/eid2309.170847

Influenza A(H3N2) Virus in Swine at Agricultural Fairs and Transmission to Humans, Michigan and Ohio, USA, 2016

Technical Appendix

Table. Influenza A virus segment sequence identifier (GenBank or GISAID EpiFlu accession no.)*

Strain Name	PB2	PB1	PA	HA	NP	NA	M	NS
A/Ohio/27/2016(H3N2)	EPI824746	EPI824747	EPI824745	EPI824749	EPI824742	EPI824748	EPI824744	EPI824743
A/Ohio/28/2016(H3N2)	EPI824754	EPI824755		EPI824757		EPI824756	EPI824752	EPI824751
A/Ohio/29/2016(H3N2)	EPI881705	EPI881706	EPI881704	EPI838255	EPI881701	EPI838254	EPI881703	EPI881702
A/Ohio/32/2016(H3N2)	EPI881724	EPI881725	EPI881723	EPI838288	EPI881720	EPI838287	EPI881722	EPI881721
A/Ohio/33/2016(H3N2)				EPI838268				
A/Ohio/35/2016(H3N2)	EPI881730	EPI881731	EPI881729	EPI838290	EPI881726	EPI838289	EPI881728	EPI881727
A/Michigan/82/2016(H3N2)	EPI824762	EPI824763	EPI824761	EPI824765	EPI824758	EPI824764	EPI824760	EPI824759
A/Michigan/83/2016(H3N2)	EPI824772	EPI824773	EPI824771	EPI824775	EPI824768	EPI824774	EPI824770	EPI824769
A/Michigan/84/2016(H3N2)	EPI838248	EPI838249	EPI838247	EPI824767	EPI838244	EPI824766	EPI838246	EPI838245
A/Michigan/87/2016(H3N2)	EPI881759	EPI881760	EPI881758	EPI838251	EPI881755	EPI838250	EPI881757	EPI881756
A/Michigan/88/2016(H3N2)	EPI881700		EPI881699	EPI838253	EPI881697	EPI838252	EPI881698	
A/Michigan/89/2016(H3N2)	EPI881710	EPI881711	EPI881709	EPI838266	EPI881707	EPI838265	EPI881708	
A/Michigan/90/2016(H3N2)	EPI838261	EPI838262	EPI838260	EPI838264	EPI838257	EPI838263	EPI838259	EPI838258
A/Michigan/91/2016(H3N2)				EPI838256				
A/Michigan/93/2016(H3N2)	EPI838272	EPI838273	EPI838271	EPI838275	EPI838269	EPI838274	EPI838270	EPI881712
A/Michigan/94/2016(H3N2)	EPI838279	EPI838280		EPI838282	EPI838276	EPI838281	EPI838277	
A/Michigan/95/2016(H3N2)				EPI838284		EPI838283		
A/Michigan/96/2016(H3N2)	EPI881718	EPI881719	EPI881717	EPI838286	EPI881714	EPI838285	EPI881716	EPI881715
A/swine/Ohio/16TOSU4783/2016(H3N2)	KX981537	KX981559	KX981486	KX981534	KX981517	KX981555	KX981506	KX981516
A/swine/Ohio/16TOSU4788/2016(H3N2)	KX981507	KX981564	KX981538	KX981496	KX981550	KX981514	KX981489	KX981549
A/swine/Michigan/16TOSU5083/2016(H3N2)	KY604999	KY605001	KY604981	KY604977	KY604983	KY604980	KY604991	KY604982
A/swine/Michigan/16TOSU5089/2016(H3N2)	KY604996	KY604987	KY604978	KY605007	KY604994	KY604990	KY604989	KY605006
A/swine/Michigan/16TOSU5222/2016(H3N2)	KY605004	KY604998	KY604988	KY604993	KY604997	KY604985	KY604986	KY605000
A/swine/Michigan/16TOSU5238/2016(H3N2)	KY604995	KY605003	KY604976	KY605005	KY604979	KY604984	KY604992	KY605002
A/swine/Ohio/16TOSU5245/2016(H3N2)	KX981522	KX981522	KX981522	KX981522	KX981522	KX981522	KX981522	KX981522
A/swine/Ohio/16TOSU5254/2016(H3N2)	KX981528	KX981562	KX981551	KX981518	KX981502	KX981536	KX981491	KX981545
A/swine/Ohio/16TOSU5873/2016(H3N2)	KX981527	KX981560	KX981487	KX981526	KX981510	KX981509	KX981548	KX981480
A/swine/Ohio/16TOSU5879/2016(H3N2)	KX981500	KX981563	KX981505	KX981554	KX981515	KX981513	KX981542	KX981520
A/swine/Ohio/16TOSU8757/2016(H3N2)	KX98148	KX981561	KX981481	KX981523	KX981492	KX981503	KX981493	KX981495
A/swine/Ohio/16TOSU8764/2016(H3N2)	KX981501	KX981566	KX981519	KX981552	KX981494	KX981553	KX981530	KX981490
A/swine/Michigan/A01104095/2016(H3N2)	KX772282	KX772283	KX772284	KX772285	KX772286	KX772287	KX772288	KX772289

*GISAID, http://platform.gisaid.org.
